# Overexpression of PBK/TOPK relates to poor prognosis of patients with breast cancer: a retrospective analysis

**DOI:** 10.1186/s12957-022-02769-x

**Published:** 2022-09-28

**Authors:** Liang Qiao, Jinling Ba, Jiping Xie, Ruiping Zhu, Yi Wan, Min Zhang, Zeyu Jin, Zicheng Guo, Jiaxuan Yu, Sijing Chen, Yongqiang Yao

**Affiliations:** 1 The Department of Breast and Thyroid Surgery, Zhongshan Hospital Affiliated to Dalian University, No. 6, Jiefang Street, Zhongshan District, Dalian, 116001 China; 2 The Pathology Department, Zhongshan Hospital Affiliated to Dalian University, Dalian, 116001 China

**Keywords:** PBK/TOPK, Overexpressed, Breast cancer, Prognosis

## Abstract

**Background:**

PDZ-binding kinase/T-lymphokine-activated killer cell-derived protein kinase (PBK/TOPK) is a potential prognostic indicator for patients with breast cancer. The objective of the present study was to explore the relationship between PBK/TOPK expression and clinicopathological indicators as well as the survival of patients with breast cancer.

**Methods:**

Immunohistochemical staining was used to detect the expression of PBK/TOPK in 202 cases of breast cancer tissues. The relationship between PBK/TOPK and clinicopathological parameters was evaluated using Spearman’s rank-order correlation. The difference in PBK/TOPK expression among different molecular types was analyzed with the chi-square test. Kaplan-Meier analysis was used to create a survival curve and the log rank test was used to analyze the overall survival (OS) and disease-free survival (DFS). Prognostic correlation was assessed using univariate and multivariate Cox regression analyses.

**Results:**

Among 202 breast cancer samples, PBK/TOPK was expressed (“+” and “++”) in 182 samples (90.1%). In addition, the histological grade, TNM stages, lymph node metastasis, estrogen receptor (ER), progesterone receptor (PR), human epidermal growth factor receptor 2 (HER-2), and Ki-67 were positively associated with PBK/TOPK expression. With regard to the molecular type, the expression of PBK/TOPK is different. The expression level of PBK/TOPK was negatively correlated with both the OS and DFS of breast cancer patients. The difference in the above results is meaningful (*P* < 0.05).

**Conclusions:**

PBK/TOPK is overexpressed in breast cancer, and the expression is closely related to the clinicopathological characteristics of the disease. Breast cancer patients with high expression of PBK/TOPK have a poor prognosis. Therefore, healthcare providers can optimize breast cancer management using this indicator.

## Background

The incidence and mortality of breast cancer rank first among female malignant cancers in China and worldwide [1, 2]. The specific causes of breast cancer are still unclear; however, some high-risk groups such as individuals with family susceptibility, hormone disorders, or immune response system disorders are more likely to develop the disease [3]. Due to the diverse biological characteristics of breast cancer, studies on the pathogenesis and etiology are conducted based on molecular biology [4].

Several critical signal transfer pathways play essential roles in breast cancer progression [4]; for example, the phosphoinositide 3-kinase/protein kinase B/mechanistic target of rapamycin (PI3K/AKT/mTOR) signaling pathway is often activated in breast cancer and plays an important role in the expansion and invasion of cancer cells [5, 6]. Human epidermal growth factor receptor 2 (HER-2) and estrogen receptor (ER)-α are upstream molecules of the PI3K/AKT/mTOR signaling pathway, which is very important for diagnosing and treating breast malignant tumors [7]. Hormone receptor-positive patients with breast cancer can be treated with endocrine therapy and have a good prognosis. On the contrary, hormone receptor-negative patients are not sensitive to endocrine therapy and have poor prognoses [8]. HER-2 is as an important predictor of breast cancer prognosis. It has been demonstrated that the overexpression of HER-2 indicates early metastasis and the rapid progression of tumors [9]. At present, clinically targeted drugs for HER-2 gene amplification are widely used clinically and have achieved definite curative effects by improving the prognosis of patients [10]. With in-depth study by researchers, new indicators that can be used as prognostic indicators for malignant tumors have been gradually uncovered.

PDZ-binding kinase/T-lymphokine-activated killer cell-derived protein kinase (PBK/TOPK) is a serine-threonine protein kinase [11]. PBK/TOPK contains 322 amino acids and belongs to the family of mitogen-activated protein kinase molecules [12], which are normally found in tissues with high proliferation potential, such as the testis and placenta, but not in normal tissues [13–15]. In recent years, studies have shown that PBK/TOPK is expressed in a variety of tumors, such as lung [16], stomach [17], and liver cancers [18, 19]. In addition, PBK/TOPK participates in many cell functions, including cell growth, DNA damage repair, the immune response, and inflammation [20, 21], and plays a vital role in cell cycle regulation and apoptosis [22]. Downregulation of the PBK/TOPK gene can cause the internal structure of a cell to become disordered and leads to a delay in cell cytokinesis during mitosis, thereby reducing the cell viability and expansion capacity and inhibiting the potential for tumor cell generation [23]. It was reported that the overexpression of PBK/TOPK protein usually indicates a poor prognosis for malignant tumors [15–18].

As a potential prognostic indicator for patients with breast cancer, PBK/TOPK has attracted increasing attention from clinicians and researchers. PBK/TOPK protein is difficult to detect in normal breast tissues but has been found in breast cancer tissues [15, 24]. In addition, PBK/TOPK may promote the proliferation of breast cancer cells by mediating the geranyl-geranylation signaling pathway [25]. However, the clinical data on PBK/TOPK in breast cancer is still limited. Therefore, the objective of the present study was to study the relationship between PBK/TOPK expression and clinicopathological indicators as well as the survival of patients with breast cancer. In addition, the influence of PBK/TOPK on the prognosis was evaluated to provide a foundation for clinical applications.

## Methods

The present study was a retrospective analysis approved by the Ethics Committee of Zhongshan Hospital Affiliated to Dalian University (Ethical Review Number: 2019302). Due to the retrospective and anonymous nature of the study, informed consent from the patients was waived.

### Patients

To collect long-term prognostic data, we screened patients diagnosed with breast cancer at the Department of Pathology from Zhongshan Hospital Affiliated with Dalian University from 1 January 2014 to 31 December 2014. The eligibility criteria for the patients were as follows: (1) patients pathologically diagnosed with invasive breast cancer; (2) no previous treatment for breast cancer; (3) the initial treatment after the diagnosis was surgery; (4) no radiotherapy, chemotherapy, or endocrine treatment was performed before the operation; (5) the postoperative specimens were available for further examination; (6) patients who completed follow-up; and (7) no history of malignancy.

### Sample size

The incidence of outcome events in the patients with breast cancer was 10.4%, and the hazard ratio of outcome events in the population with high PBKTOPK was 4.59 compared with the general population. In the case of test level = 0.05 and power = 0.9, a sample size of 183 cases was required. Consider 10% lost to follow-up, and 192 cases were finally needed.

### Data collection

We collected the basic clinicopathological characteristics of the patients including the age, menopausal status, tumor size, TNM stage following the AJCC TNM 8th edition [26], histological grade, lymph node metastasis, number of lymph node metastases, vascular tumor thrombus, ER, progesterone receptor (PR), HER-2, and the nuclear protein Ki67. The exposure factor was the localization of PBK/TOPK protein in the nucleus or cytoplasm. The primary outcomes were overall survival (OS) and disease-free survival (DFS). OS was defined as the period from the day of the operation to the day of death for any reason or at the end of follow-up. The DFS was defined as the period from the day of the operation to the day of any disease progression (recurrence or metastasis) or the end of follow-up.

### Evaluation of the PBK/TOPK expression

All paraffin tissue sections from the included patients were collected at the Zhongshan Hospital Affiliated to Dalian University. PBK/TOPK antibody was purchased from Abcam (dilution 1:4000). Two pathologists with greater than 5 years of experience evaluated and scored the PBK/TOPK expression.

The standard procedures for preparing and evaluating PBK/TOPK expression were as follows: tissue sections were placed in a constant temperature oven at 60 °C for 3 h. After dewaxing and hydration, the antigen was repaired via placement in a microwave oven three times for 3 min with intervals of 5 min (3 min, 5 min, 3 min, 5 min, 3 min). Subsequent procedures consisted of incubation with a blocking solution of endogenous peroxidase at room temperature for 20 min, incubation using a working solution of blocking goat serum at room temperature for the next 20 min, incubation with the PBK/TOPK antibody in a refrigerator at 4 °C overnight for 12 h, incubation with biotin-labeled goat anti-rabbit IgG polymer at room temperature for 30 min, incubation with horseradish enzyme-labeled streptomycin at room temperature for 30 min, color development using 3,3′-diaminobenzidine (DAB) reagent after washing the slides with phosphate-buffered saline (PBS), and re-staining with hematoxylin for 15 s. Finally, the slides were dehydrated, cleared, sealed, read, and recorded.

**Fig. 1:**
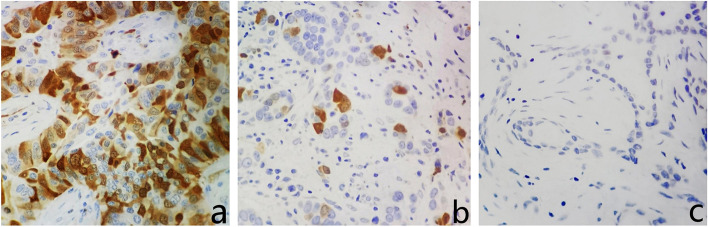
PBK/TOPK protein expression in breast cancer tissues (original magnification ×400). **A** High expression of PBK/TOPK. **B** Low expression of PBK/TOPK. **C** No expression of PBK/TOPK

PBK/TOPK protein was localized in the nucleus or cytoplasm. Five fields of view for each patient were randomly selected under 400× objective lens for scoring. First, the color of the rendering was scored (colorless (0 points), pale-yellow (1 point), brownish-yellow (2 points), and brown (3 points). Then, the color rendering ratio was calculated as follows: 1–10% for 1 point, 11–50% for 2 points, 51–80% for 3 points, and > 80% for 4 points. Next, the immunization score was generated by multiplying the above two scores. Furthermore, 0 points, 1–4 points, and > 4 points were marked as “−,” “+,” and “++,” respectively (Fig. 1). Finally, using 4 points as the cutoff value, we divided the expression of PBK/TOPK in breast cancer into two groups, with a score ≤ 4 indicating low expression and a score > 4 indicating high expression [27].

### Statistical analysis

SPSS 20.0 software was used to analyze the data in the present study. Categorical variables were described as counts and percentages, while continuous variables were expressed as the mean and standard deviation (SD). The relationship between PBK/TOPK and clinicopathological parameters was evaluated with the Spearman’s rank-order correlation. The difference in PBK/TOPK expression among different molecular types was evaluated with the chi-square test. Kaplan-Meier analysis was used to generate survival curves, and the log-rank test was used to analyze OS and DFS. Prognostic correlation analysis was performed using univariate and multivariate Cox regression analyses. The receiver operating characteristic (ROC) curve was drawn to determine the sensitivity and specificity of PBK/TOPK for OS. All of the above analyses were performed with a threshold of *P* < 0.05 (two-sided) for statistical significance.

## Results

### Characteristics of the included patients

**Fig. 2:**
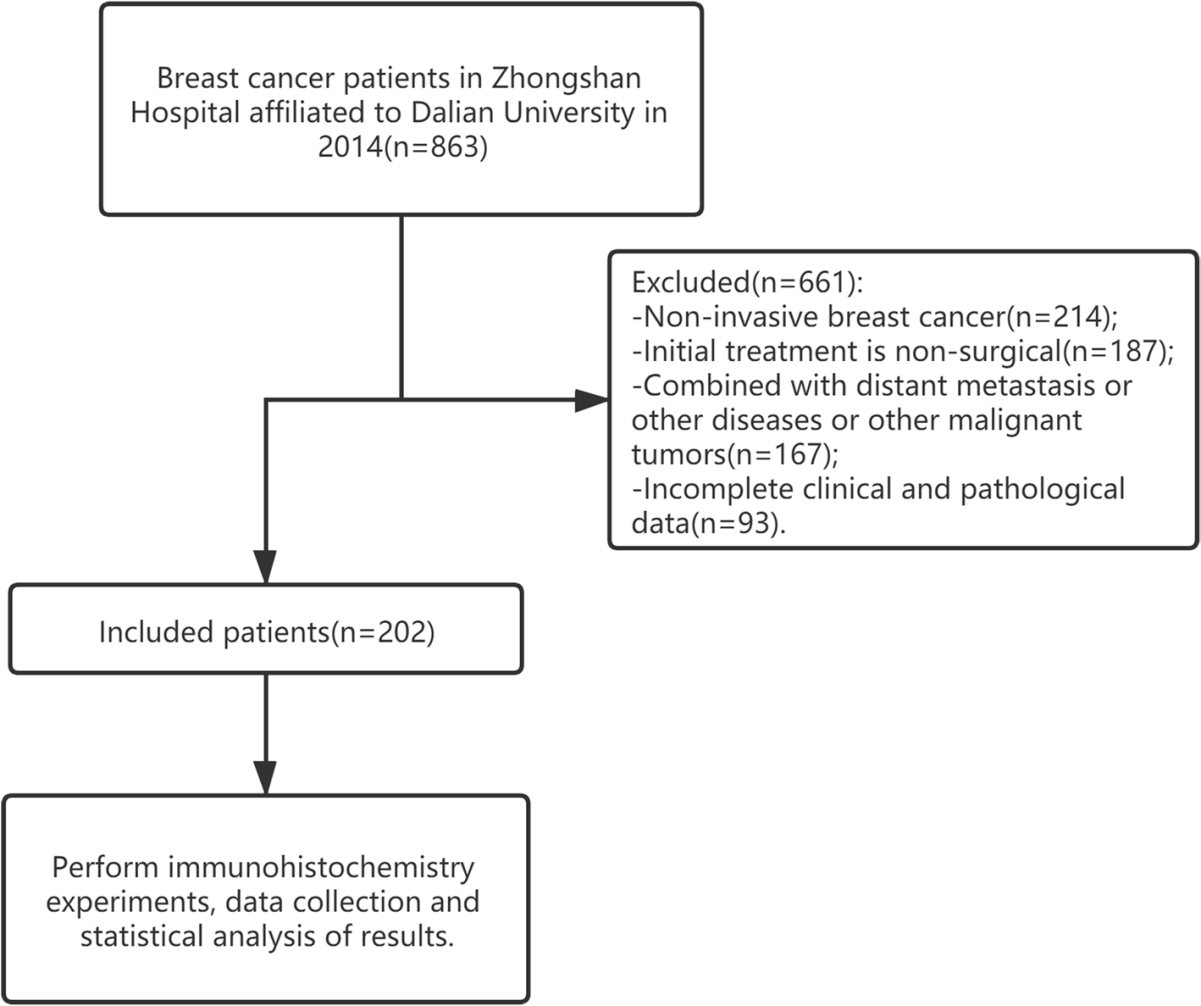
Flow chart of the study screening process

We screened 863 patients with breast cancer treated in 2014 at our hospital. According to our eligibility criteria, 202 patients were included in the final analysis. The workflow for the screening processes is illustrated in Fig. 2. During the study, there were 6.4% (13/202) patients were lost to follow-up.

The mean age of the included patients was 52.41 ± 10.97 (mean ± SD). Most of the tumors (191/202, 94.6%) were ≤ 5 cm. Forty-one patients (20.3%) had TNM stage 3 disease. The details of the baseline pathological characteristics are listed in Table 1.

**Table 1:** The clinicopathological characteristics of included patients and their correlations with the expression of PBK/TOPK

Parameter	Total, ***N*** (%)	PBK/TOPK			
	(***n*** = 202)	Low, ***N*** (%)	High, ***N*** (%)	***r***_**s**_	***P***
**Age**					
≤ 50 y	94 (46.5)	52 (55.3)	42 (44.7)	−0.049	0.487
> 50 y	108 (53.5)	65 (60.2)	43 (39.8)		
**Menopausal state**					
Premenopausal	115 (56.9)	63 (54.8)	52 (45.2)	−0.073	0.301
Postmenopausal	87(43.1)	54 (62.1)	33 (37.9)		
**Tumor size**					
≤ 2 cm	95 (47.0)	61 (64.2)	34 (35.8)		
> 2 cm ≤ 5 cm	96 (47.6)	51 (53.1)	45 (46.9)	0.124	0.078
> 5 cm	11 (5.4)	5 (45.5)	6 (54.5)		
**Histological**					
G1	20 (9.9)	13 (65.0)	7 (35.0)		
G2	147 (72.8)	93 (63.3)	54 (36.7)	0.171	0.015*
G3	35 (17.3)	11 (31.4)	24 (68.6)		
**TNM**					
I	66 (32.7)	45 (68.2)	21 (31.8)		
II	95 (47.0)	54 (56.8)	41 (43.2)	0.175	0.013*
III	41 (20.3)	18 (43.9)	23 (56.1)		
**Cancer thrombus**					
No	172 (85.1)	103 (59.9)	69 (40.1)	0.095	0.178
Yes	30 (14.9)	14 (46.7)	16 (53.3)		
**Lymph node metastasis**					
No	113 (55.9)	76 (67.2)	37 (32.8)	0.213	0.002*
Yes	89 (44.1)	41 (46.1)	48 (53.9)		
**Number of lymph nodes**					
1–3	56 (27.7)	29 (51.8)	27 (48.2)		
4–9	20 (9.9)	10 (50.0)	10 (50.0)	0.241	0.001*
≥ 10	13 (6.5)	2 (15.4)	11 (84.6)		
**Estrogen receptor**					
Negative	53 (26.2)	22 (41.5)	31 (58.5)	−0.198	0.005*
Positive	149 (73.8)	95 (63.8)	54 (36.2)		
**Progesterone receptor**					
Negative	110 (54.5)	56 (50.9)	54 (49.1)	−0.155	0.027*
Positive	92 (45.5)	61 (66.3)	31 (33.7)		
**HER-2**					
Negative	137 (67.8)	85 (62.0)	52 (38.0)	0.153	0.037*
Positive	49 (24.2)	22 (44.9)	27 (55.1)		
**Ki-67**					
Low expression	65 (32.2)	52 (80.0)	13 (20.0)	0.312	< 0.001*
High expression	137 (67.8)	64 (46.7)	73 (53.3)		
**Molecular typing**					
Luminal A	33 (16.3)	30 (90.9)	3 (9.1)	22.031^	< 0.001^
Luminal B	74 (36.6)	43 (58.1)	31 (41.9)		
HER-2(+)	49 (24.2)	22 (44.9)	27 (55.1)		
TNBC	30 (14.9)	12 (40.0)	18 (60.0)		

*Spearman correlation statistical analysis

^Chi-square test

### The expression of PBK/TOPK protein

Among 202 breast cancer tissues, PBK/TOPK protein was expressed (“+” and “++”) in 182 (90.1%) in Table 2. According to our criteria, high expression and low expression of PBK/TOPK were found in 85 (42.1%) and 117 (57.9%) samples, respectively.

**Table 2:** PBK/TOPK expression in breast cancer tissues

PBK/TOPK	*n*	%
(−)	20	9.9
(+)	97	48.0
(++)	85	42.1
High	85	42.1
Low	117	57.9

### Correlation between PBK/TOPK protein expression and clinicopathological characteristics

According to the Spearman correlation analysis, histological grade (*P* = 0.015), TNM stages (*P* = 0.013), lymph node metastasis (*P* = 0.002), low ER (*P* = 0.005), low PR (*P* = 0.027), HER-2 (*P* = 0.037), and Ki-67 (*P* < 0.001) were associated with PBK/TOPK protein expression. However, the patients’ age (*P* = 0.431), menopausal status (*P* = 0.163), tumor size (*P* = 0.163), and vascular tumor thrombus (*P* = 0.110) had no significant correlation with PBK/TOPK protein expression. The detailed data for the correlation analysis are presented in Table 1.

**Fig. 3:**
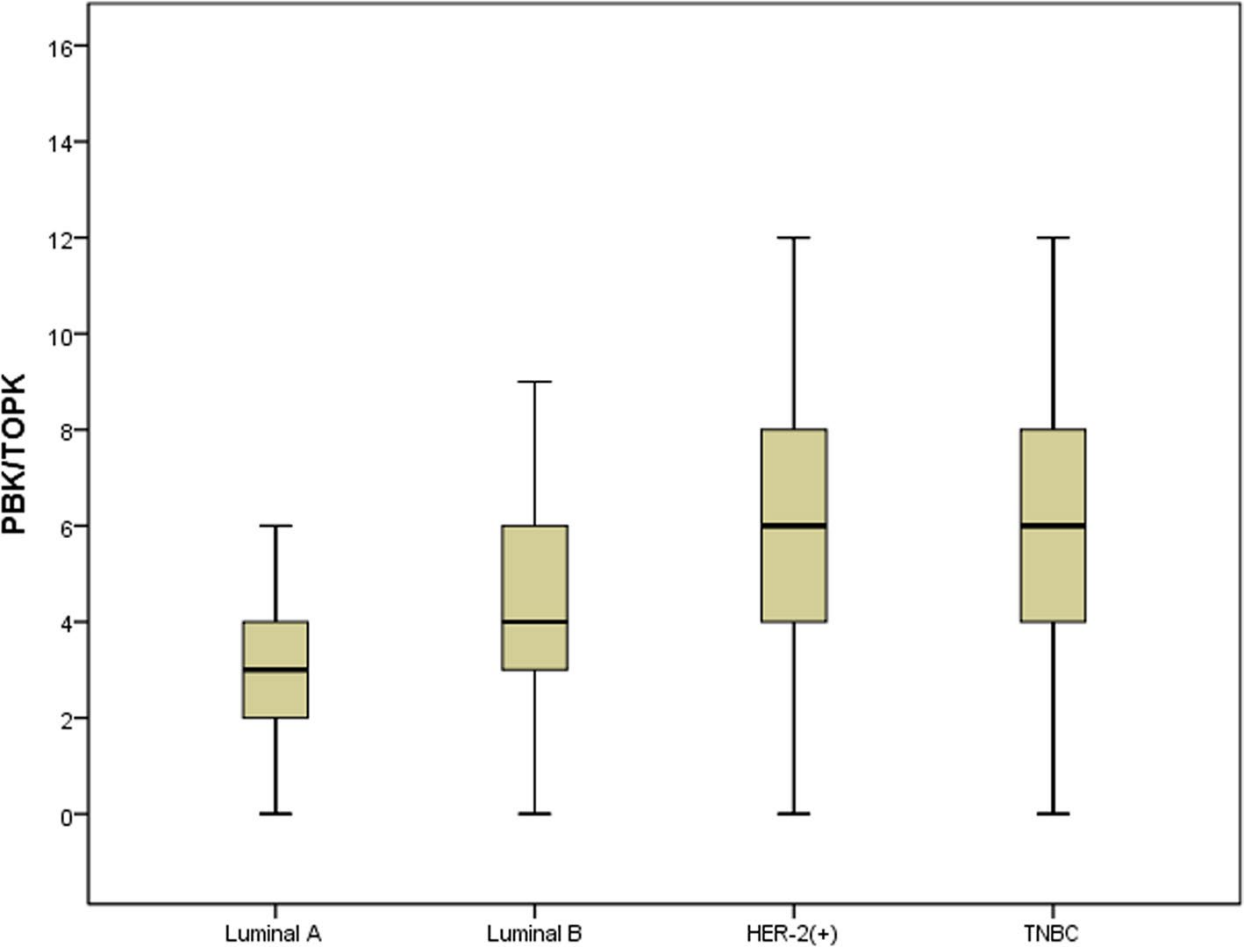
Box plot of PBK/TOPK expression in molecular typing of breast cancer

In terms of the molecular types, high expression of PBK/TOPK was observed in 9.10% (3/33), 41.88% (31/74), 55.1% (27/49), and 60.00% (18/30) of patients with luminal A type, luminal B type, HER-2(+) type, and triple negative breast cancer (TNBC), respectively (*P* < 0.001, Table 1). According to the box plots (Fig. 3), the median expression level of PBK/TOPK in luminal A samples was lower than that in luminal B samples. Additionally, the median expression levels of PBK/TOPK in luminal A and luminal B samples were lower than those of HER-2(+) and TNBC.

### PBK/TOPK protein expression level and prognosis

The follow-up for all included patients ended on 31 December 2020, and 21 patients died due to breast cancer during this period. In the study, all 21 dead patients developed distant metastasis, including bone metastasis of different degrees in all cases, four cases of chest wall + lung + thoracic metastases, two patients of lung + thoracic metastases, five patients of liver + abdominal metastasis, six cases of lung + liver metastasis, three cases of brain metastasis + lung + liver, and one case of abdominal + kidney + pancreas metastasis. The median follow-up was 78 months (ranging from 72 to 83 months). The median OS and DFS were not calculated during the study period.

**Fig. 4:**
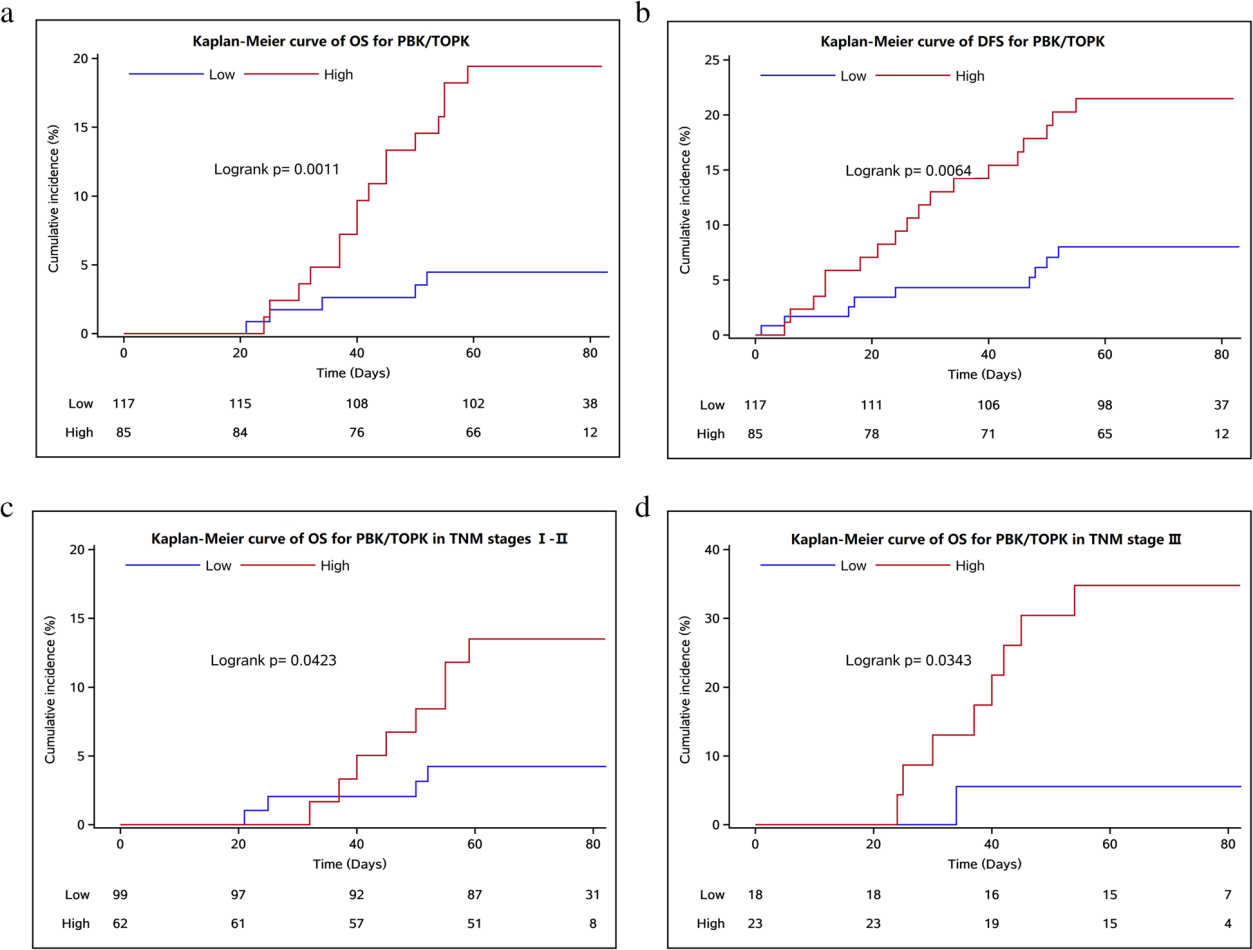
Kaplan-Meier curve of **A** OS for PBK/TOPK. **B** DFS for PBK/TOPK. **C** OS for PBK/TOPK in TNM stages I–II. **D** OS for PBK/TOPK in TNM stage 3

Kaplan-Meier analysis revealed that the expression level of PBK/TOPK was negatively correlated with the OS of breast cancer patients. The incidence of events with high PBK/TOPK expression increased significantly with prolonged follow-up (Fig. 4A). According to the log-rank test, breast cancer patients with high PBK/TOPK expression had a poor prognosis (*P* = 0.001). Similarly, the expression level of PBK/TOPK was negatively correlated with the DFS of the patients. With prolonged follow-up, the incidence of recurrent and metastatic events with high PBK expression increased significantly (Fig. 4B). The log-rank test showed that breast cancer patients with high PBK/TOPK expression had poor prognoses (*P* = 0.006).

Subgroup analysis was performed for OS according to the TNM stages using Kaplan-Meier analysis. For patients with stages 1–2 breast cancer, the expression level of PBK/TOPK was negatively correlated with OS. The incidence of events with high PBK/TOPK expression increased significantly with prolonged follow-up (Fig. 4C). According to the log-rank test, breast cancer patients with high PBK/TOPK expression had poor prognoses (*P* = 0.042). For patients with stage 3 breast cancer, the expression level of PBK/TOPK was negatively correlated with OS as well. The incidence of events with high PBK/TOPK expression increased significantly with prolonged follow-up (Fig. 4D). According to the log-rank test, breast cancer patients with high PBK/TOPK expression had poor prognoses (*P* = 0.034).

### Influential factors related to the prognosis

Univariate Cox regression analysis revealed that tumor size (OS: hazard ratio (HR) = 4.148, *P* = 0.010; DFS: *HR* = 3.452, *P* = 0.007), lymph node metastasis (OS: *HR* = 3.390, *P* = 0.012; DFS: *HR* = 2.351, *P* = 0.032), HER-2 (OS: *HR* = 2.016, *P* = 0.002; DFS: *HR* = 1.977, *P* = 0.001), and PBK/TOPK (OS: *HR* = 4.590, *P* = 0.003; DFS: *HR* = 2.890, *P* = 0.009) were risk factors for both OS and DFS among the included patients. On the contrary, ER expression (OS: *HR* = 0.342, *P* = 0.014; DFS: *HR* = 0.401, *P* = 0.018) was a protective factor for the OS and DFS of the patients. Furthermore, the TNM stage (OS: *HR* = 2.658, *P* = 0.030) was a risk factor for the OS of the patients. The results of the univariate analysis are listed in Table 3.

**Table 3:** Univariate Cox regression analysis of OS and DFS for all included patients

Variable	OS			DFS		
	HR	95% ***CI***	***P***	HR	95% ***CI***	***P***
**Age** (≤ 50 y vs. > 50 y)	1.502	0.623–3.624	0.365	1.563	0.716–3.413	0.263
**Menopausal state** (premenopausal vs Postmenopausal)	0.781	0.342–1.884	0.582	0.887	0.412–1.912	0.760
**Tumor size** (≤ 2 cm vs > 2 cm)	4.148	1.395–12.330	0.010*	3.452	1.393–8.557	0.007*
**Histological grade** (G1, G2, or G3)	2.020	0.784–5.208	0.145	1.812	0.766–4.285	0.176
**TNM stage** (I, II, vs III)	2.658	1.101–6.414	0.030*	2.195	0.986–4.887	0.054
**Cancer thrombus** (no vs yes)	2.505	0.972–6.457	0.057	1.829	0.738–4.533	0.192
**Lymph node metastasis** (no vs yes)	3.390	1.315–8.742	0.012*	2.351	1.076–5.136	0.032*
**Estrogen receptor** (negative vs positive)	0.342	0.145–0.806	0.014*	0.401	0.187–0.856	0.018*
**Progesterone receptor** (negative vs positive)	0.443	0.172–0.142	0.092	0.671	0.307–1.465	0.317
**HER-2** (negative vs positive)	2.016	1.283–3.168	0.002*	1.977	1.328–2.943	0.001*
**Ki-67** (low vs high)	2.105	0.708–6.257	0.180	2.146	0.812–5.666	0.123
**PBK/TOPK** (low vs high)	4.590	1.681–12.530	0.003*	2.890	1.298–6.433	0.009*
**Chemotherapy** (yes vs no)	2.003	0.268–15.018	0.498	2.669	0.361–19.728	0.336

*Cox ratio univariate analysis is statistically significant (*P* < 0.05)

**Fig. 5:**
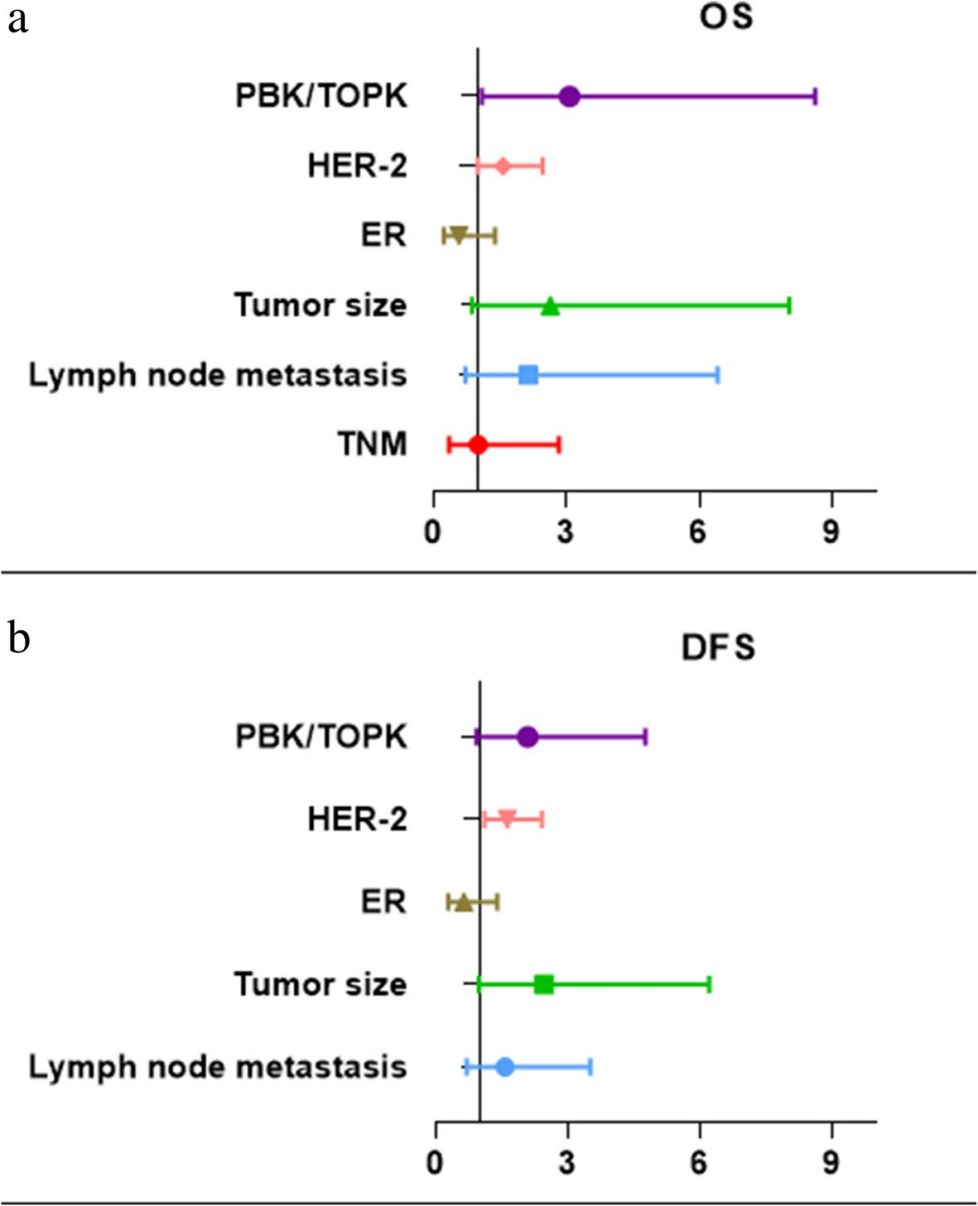
Forest plot for multivariate Cox regression analysis of OS and DFS for all included patients. The multivariate Cox analysis results of OS and DFS show that PBK/TOPK and HER2 were statistically significantly associated with OS. In addition, only HER2 was statistically significantly associated with DFS

**Fig. 6:**
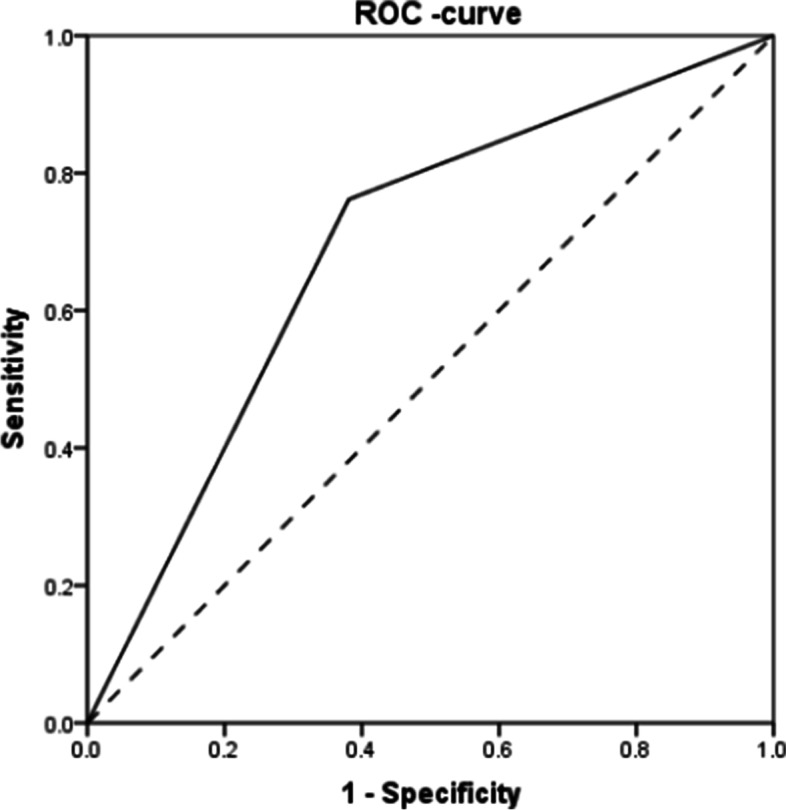
The ROC curve of PBK/TOPK overexpression for prediction of overall survival. The ROC curve combines the sensitivity and specificity of PBK/TOPK overexpression for breast cancer diagnosis. The closer the curve is to the upper left corner, the larger is the area under the curve, indicating greater diagnostic value. As the ROC curve shows, the experimental ROC curve is close to the upper left corner, and the area under the curve is 0.690 > 0.5, indicating that the model has a predictive value

The meaningful indicators generated from the univariate analysis (*P* < 0.05) were included in the multivariate Cox regression, and the results are presented in Table 4 and Fig. 5. High expression of PBK/TOPK was an independent influential factor on OS in breast cancer (*P* = 0.034), and HER-2 expression was an independent influential factor on DFS (*P* = 0.014). Analysis of the ROC curve in Fig. 6 revealed that the area under the curve was 0.690 (95% confidence interval (CI): 0.576–0.805) for the expression of PBK/TOPK in the prediction of 5-year OS (*P* = 0.004).

**Table 4:** Multivariate Cox regression analysis of OS and DFS for all included patients

Variable	OS			DFS		
	HR	95% ***CI***	***P***	HR	95% ***CI***	***P***
**TNM** (I, II, or III)	1.002	0.355–2.829	0.997			
**Lymph node metastasis** (no vs yes)	2.139	0.714–6.408	0.174	1.574	0.705–3.513	0.268
**Tumor size** (≤ 2 cm vs > 2 cm)	2.636	0.865–8.026	0.088	2.461	0.975–6.212	0.057
**ER** (negative vs positive)	0.572	0.236–1.388	0.217	0.636	0.289–1.398	0.260
**HER-2** (negative vs positive)	1.566	0.996–2.464	0.052	1.633	1.104–2.416	0.014*
**PBK/TOPK** (low vs high)	3.065	1.091–8.610	0.034*	2.091	0.918–4.764	0.079

*Cox ratio multivariate analysis is statistically significant (*P* < 0.05)

## Discussion

Breast cancer is a type of malignant tumor with high heterogeneity. Different molecular types of breast cancer have different clinical manifestations [28]. In this retrospective study, we explored PBK/TOPK protein expression in patients with breast cancer. PBK/TOPK protein is difficult to detect in normal tissues but has been found in a variety of malignant tumors [15]. Among 202 breast cancer samples, high expression of PBK/TOPK was found in 85 cases (42.1%), consistent with a previous report (104/290, 35.9%) by O'Leary et al. [29]. This experiment confirmed the overexpression of PBK/TOPK in breast cancer tissues.

In clinical practice, lymph node metastasis, the number of lymph nodes with metastasis, histological grade, TNM stage, ER, PR, HER-2, and Ki-67 are traditional prognostic indicators for patients with breast cancer [30–33]. In the present study, PBK/TOPK protein expression was positively associated with the risk factors for prognosis, including histological grade (*P* = 0.015), TNM stages (*P* = 0.013), lymph node metastasis (*P* = 0.002), HER-2 (*P* = 0.037), and Ki-67 (*P* < 0.001) but negatively associated with the beneficial factors ER (*P* = 0.005) and PR (*P* = 0.027). The results suggest that the expression level of PBK is related to the prognosis of breast cancer. According to our analysis, when the histology and TNM stage are higher, with lymph node metastasis and HER-2 positivity, the positive expression of PBK/TOPK is more prominent, and the prognosis is relatively poor. For instance, in the present study, higher TNM stages corresponded with higher PBK/TOPK expression and worse prognosis. As the prognosis of stage 3 breast cancer patients was worse than those of patients in stages 1 and 2, the high expression of PBK/TOPK indicated that it was associated with the proliferation of malignant tumors and tumor progression, which was consistent with the existing evidence [25].

Furthermore, according to our survival analysis, the expression level of PBK/TOPK was negatively correlated with both the OS and the DFS of breast cancer patients. Using multivariate Cox regression analysis, we confirmed that PBK/TOPK was an independent risk factor for the prognosis of patients with breast cancer (*P* = 0.034) (Fig. 5). The finding that PBK/TOPK protein overexpression predicts poor prognosis for patients with breast cancer is consistent with a previous study [29] and other prognostic studies on other malignancies [15–18, 34, 35]. The OS and DFS of breast cancer patients with high expression of PBK/TOPK are shortened in the present study, indicating that PBK/TOPK expression is correlated with the prognosis of malignant tumors. The prognosis of patients with high expression of PBK/TOPK is worse.

In 2000, Perou et al. proposed a molecular classification system for breast cancer, comprising luminal A, luminal B, HER-2 overexpression, and basal-like type (including TNBC) [36]. Luminal A has the best prognosis, whereas basal-like type breast cancer has the worst prognosis. Carey et al. followed 496 breast cancer patients for 8.1 to 11.2 years and concluded that the prognoses for patients with HER-2 overexpression and TNBC were the worst, whereas luminal A had the best prognosis [37]. According to our analysis, the overexpression of PBK/TOPK in luminal A was significantly lower than that of HER-2(+) and TNBC types, which once again confirmed that PBK/TOPK overexpression is associated with a worse prognosis for breast cancer. In breast cancer molecular typing, luminal A and luminal B breast cancers have better prognosis than HER-2 positive and TNBC breast cancers, and the expression of PBK/TOPK in HER-2 positive and TNBC is higher than that in luminal A or luminal B types. In luminal types of breast cancer, ER and PR hormone receptors are positive, and the expression of PBK/TOPK is relatively low. In contrast, ER and PR hormone receptors are negative in HER-2 positive and TNBC breast cancer, the expression of PBK/TOPK is higher, and the expression level of PBK/TOPK is negatively correlated with the positive expression of ER and PR. This phenomenon may suggest that PBK/TOPK inhibits the expression of ER or PR, or that the expression of ER or PR inhibits the expression of PBK/TOPK.

To the best of our knowledge, this study is the first investigation using clinical data to explore the expression of PBK/TOPK in breast cancer and its relationship with patient prognosis. We conducted several rigorous statistical tests, including correlation tests, Kaplan-Meier curves, and Cox regression analysis, to confirm our findings. There are several limitations. First, the sample size for the study was relatively small. Additionally, not all specimens could be used in the study. Only slides stained immunohistochemically to detect the expression of PBK/TOPK protein in paraffin tissue samples of breast cancer were included in the final analysis. Next, due to the inherent characteristics of observational study, some potential biases cannot be avoided; for instance, 13 patients were lost to follow-up, which can lead to missing information bias. Large-scale prospective studies are needed to confirm our findings in the future. Third, as the present study was a retrospective study using the data from our unit, we did not register it in any public databases or clinical trial registries beforehand. In addition, we found that PBK/TOPK protein was expressed in both the nucleus and the cytoplasm. Therefore, one of our research plans in the future is to study the difference in clinical impact between breast cancer in which PBK/TOPK is mainly expressed in the nucleus of cancer cells and that in which it is mainly expressed in the cytoplasm of the cancer cells.

## Conclusion

With regard to the practical implications, this study provides evidence that PBK/TOPK protein is overexpressed in breast cancer tissues and predicts a poor prognosis for patients with breast cancer, which can provide clinicians with diagnosis and treatment value.

PBK/TOPK protein is overexpressed in breast cancer, and its expression is closely related to the clinicopathological characteristics of the disease. Breast cancer patients with high expression of PBK/TOPK have a poor prognosis. Therefore, healthcare providers can optimize breast cancer management using this indicator.

## Data Availability

The datasets used and/or analyzed during the current study are available from the corresponding author on reasonable request.

## Abbreviations

PBK: PDZ-binding kinase
TOPK: T-LAK cell-originated protein kinase
ER: Estrogen receptor
PR: Progesterone receptor
HER-2: Human epidermal growth factor receptor-2
Ki-67: Nuclear-associated antigen Ki-67
OS: Overall survival
DFS: Disease-free survival
PBS: Phosphate-buffered saline
DAB: 3, 3N-Diaminobenzidine
